# Engineering approaches for understanding mechanical memory in cancer metastasis

**DOI:** 10.1063/5.0194539

**Published:** 2024-04-10

**Authors:** Jia Wen Nicole Lee, Andrew W. Holle

**Affiliations:** 1 Mechanobiology Institute, National University of Singapore, 117411 Singapore, Singapore; 2 Department of Biomedical Engineering, National University of Singapore, 117411 Singapore, Singapore

## Abstract

Understanding cancer metastasis is crucial for advancing therapeutic strategies and improving clinical outcomes. Cancer cells face dynamic changes in their mechanical microenvironment that occur on timescales ranging from minutes to years and exhibit a spectrum of cellular transformations in response to these mechanical cues. A crucial facet of this adaptive response is the concept of mechanical memory, in which mechanosensitive cell behavior and function persists even when mechanical cues are altered. This review explores the evolving mechanical landscape during metastasis, emphasizing the significance of mechanical memory and its influence on cell behavior. We then focus on engineering techniques that are being utilized to probe mechanical memory of cancer cells. Finally, we highlight promising translational approaches poised to harness mechanical memory for new therapies, thereby advancing the frontiers of bioengineering applications in cancer research.

## I. INTRODUCTION

Cancer is a devastating disease that continues to challenge the medical community, in large part due to its ability to metastasize to diverse and distant tissues. In conjunction with extensive research on both the biology of cancer progression and clinical approaches to treatment, the role of mechanical cues encountered during the metastatic journey has emerged as a crucial avenue to tackle this disease. For example, knowledge that tumor tissue tends to be stiffer than healthy tissue has led to the widespread public health education of breast self-examination and the development of lysyl oxidase inhibitor drugs as anti-stromal therapy that targets the cross-linking of extracellular matrices (ECM) (Setargew *et al.*, 2021). Metastasis begins at the boundary of the primary tumor when cells invade into the surrounding stromal tissue. They then enter the circulatory system via a transendothelial migration known as intravasation. These cells, also known as circulating tumor cells (CTCs), lose their cell–ECM attachments and are carried by the circulatory flow throughout the body. Finally, in distant capillary beds, they extravasate into a new tissue niche where they begin to form metastatic colonies. During each step of this process, cells are exposed to diverse physical signals capable of driving invasiveness and increasing their mutational burden.

In the past decade, a novel and promising concept known as “mechanical memory” has emerged in the field of cellular mechanobiology. This phenomenon was first described in 2012 with primary rat lung fibroblasts that had been cultured on stiff substrates for three weeks, causing them to differentiate into myofibroblasts characterized by α-smooth muscle actin and enhanced contractility. After transferring these mechanically primed cells to soft substrates, they persisted in the myofibroblast state for up to two weeks, proving that the integration of mechanical signals can lead to irreversible long-term changes in cell state, even after removal of the mechanical stimulus (Balestrini *et al.*, 2012). Subsequently, mechanical memory was identified in human mesenchymal stem cells, which were found to remember their past physical signals when grown on soft hydrogels. This activation led to persistent chromatin remodeling, upregulation of Yes-associated protein (YAP), and the pre-osteogenic transcription factor RUNX2, with differential expression based on the duration of their previous culture on significantly stiffer culture plates (Killaars *et al.*, 2019; Yang *et al.*, 2014). Similar to fibroblasts and stem cells, cancer cells being migratory cells that experience a multitude of different mechanical cues at different points of the metastatic cascade may possess the ability to retain the behaviors and functions influenced by past microenvironments, thereby influencing their future fate during the metastatic journey. Cancer research has long paid close attention to mechanical cues at the primary tumor site and has shown that mechanical conditioning can drive tumor progression via memory of the ECM (DuFort *et al.*, 2011). In recent years, multiple research groups have provided comprehensive reviews on the mechanisms underlying mechanical memory, highlighting the relevance and exciting nature of mechanical memory in the field of mechanobiology (Beedle and Roca-Cusachs, 2023; Cambria *et al.*, 2024; Dudaryeva *et al.*, 2023; Kanoldt *et al.*, 2019; Lele *et al.*, 2020; and Scott *et al.*, 2023). Unraveling the dynamics of mechanical memory within the tumor microenvironment is an exciting new approach to understanding how cancer cells adapt and evolve, offering the potential for the development of targeted therapeutic strategies aimed at disrupting or leveraging these mechanical cues to impede or even reverse the malignant progression of the disease.

**FIG. 1.**
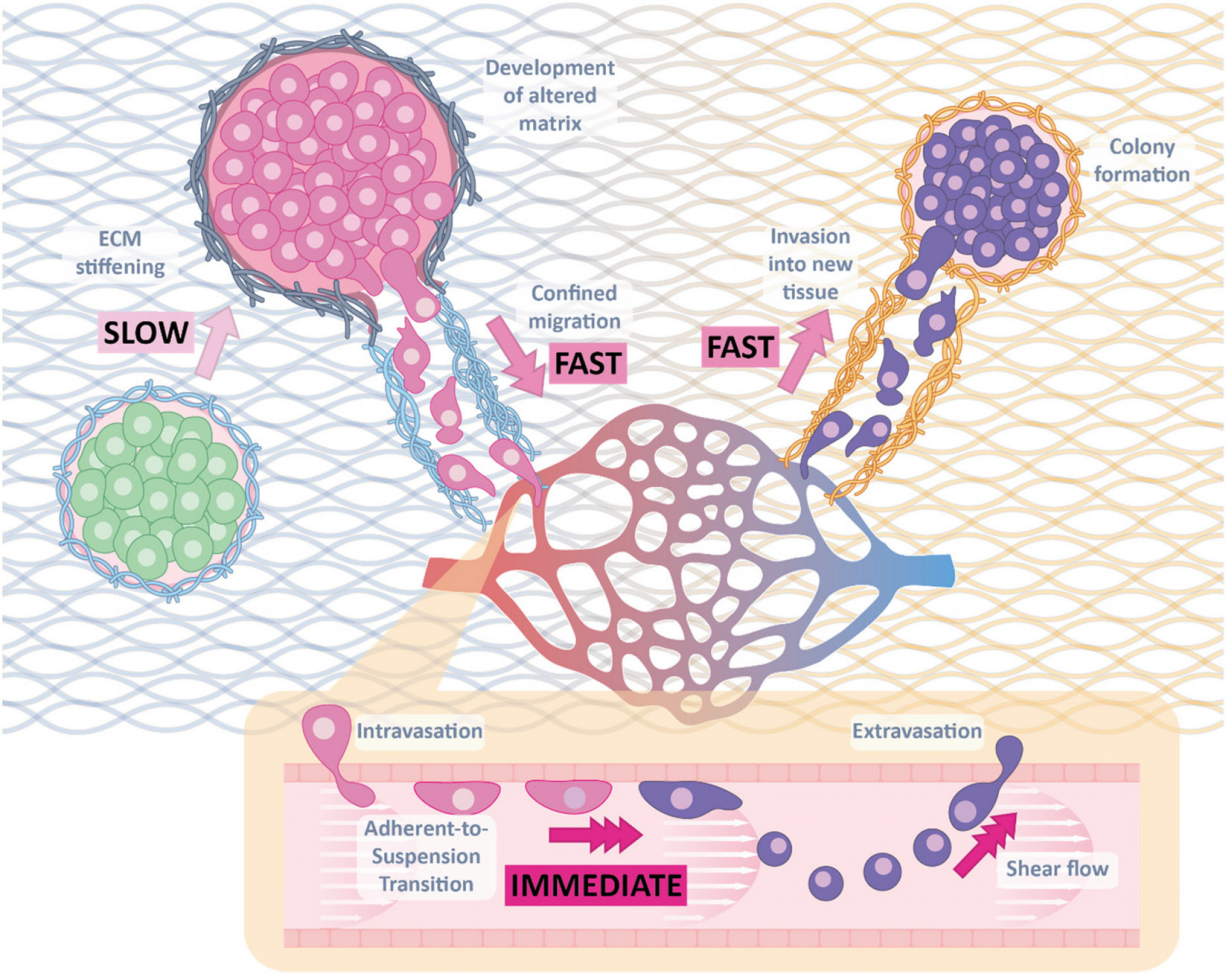
Time scales of mechanical changes during cancer metastasis. Dynamic changes of the mechanical microenvironment during cancer metastasis occur at different time resolutions. Key processes, such as ECM stiffening, occur slowly, over the course of months to years, in comparison with fast processes such as confined migration and immediate processes like the adherent-to-suspension transition.

In this review, we delve into multifaceted findings pertaining to mechanical memory in the context of cancer metastasis, with a specific emphasis on the intricate roles played by mechanical forces during the metastatic journey. By exploring the dynamic interplay between the tumor microenvironment's mechanical cues and the adaptive responses of cancer cells, we aim to shed light on the influence of these mechanical cues on the progression of metastasis and subsequently how these adaptations are retained as mechanical memory (Fig. 1). Additionally, we assess recent engineering approaches that have revolutionized the ability to test and manipulate mechanical memory. Finally, we explore the budding translational potential of harnessing this knowledge for the development of therapeutic strategies that leverage the concept of mechanical memory, thus paving the way for novel mechanotherapy approaches in the ongoing battle against cancer.

## II. NO PLACE LIKE HOME: MECHANICAL FORCES IN THE PRIMARY NICHE

The initiation of the metastatic cascade commences within the primary niche, characterized by the accumulation of mutations that promote subsequent cellular migration and metastasis to a secondary niche. At this primary niche, the mechanical microenvironment undergoes many dynamic changes that promote the invasion of cancer cells.

A well-characterized alteration of the microenvironment within the primary niche is a significant elevation in matrix stiffness. As cancerous lesions progress over periods ranging from months to years, the extracellular matrix (ECM) undergoes significant remodeling by both cancer cells and cancer associated fibroblasts (García-Palmero *et al.*, 2016; Pankova *et al.*, 2019). This results in the deposition of fibrous ECM proteins and increased collagen and elastin cross-linking facilitated by lysyl oxidase (Levental *et al.*, 2009). Cumulatively, this drives an overall stiffening of the tissue. Studies across various cancer types, including breast, lung, skin, and liver cancer, have highlighted this phenomenon, revealing stiffness increases of up to sixfold (Lopez *et al.*, 2011; Masuzaki *et al.*, 2007; Miyazawa *et al.*, 2018; Plodinec *et al.*, 2012; and Troyanova-Wood *et al.*, 2019). The correlation between heightened matrix stiffness and breast cancer risk has been recognized since 1976, with studies indicating a four- to sixfold increased risk of tumor development associated with increased breast epithelium stiffness (Wolfe, 1976). Increased stiffness of the tumor is also associated with a higher genome variation and tumor mutational burden (Pfeifer *et al.*, 2017). The implications of this stiffer microenvironment on tumor cell behavior have also been explored, with some groups finding that it is sufficient to trigger epithelial–mesenchymal transition and enhanced integrin signaling, which together promote the development of a more invasive phenotype and an increase in proliferation by regulating mechanosensitive transcription factors (Dong *et al.*, 2019; Fattet *et al.*, 2020; Ishihara and Haga, 2022; and Levental *et al.*, 2009).

As cells remodel the ECM at the primary tumor site, the organization and topography of the surrounding tissue also undergoes dynamic changes. In healthy tissue, collagen fibers are arranged in a random isotropic pattern. During tumor advancement, fibers are progressively rearranged into a parallel anisotropic orientation (Goetz *et al.*, 2011). Cancer-associated fibroblasts alter the tumor stroma by contributing to this reorganization of the ECM and the formation of parallel tracks of collagen fibers (Amatangelo *et al.*, 2005; Friedl and Wolf, 2008). Cancer cells orient themselves along these distinct matrix arrangement, generating anisotropic force via adhesion and cytoskeletal complexes that drive contact guided migration, ultimately leading to the initiation of local invasion into healthy tissue surrounding the solid tumor (Gaggioli *et al.*, 2007; Kim *et al.*, 2021; Provenzano *et al.*, 2006; Ray and Provenzano, 2021; and Seo *et al.*, 2015).

In addition to matrix remodeling, primary tumor cells also experience a high degree of mechanical stretching at the tumor boundary due to rapid proliferation of the constituent cells. Modeling and experimental approaches have shown that this stretching can drive YAP/TAZ activation, as well as the reactivation of the cell cycle in contact-inhibited cells (Aragona *et al.*, 2013). This YAP/TAZ activity is distinct from canonical mechanosensitive YAP/TAZ nuclear translocation and instead occurs via Wnt or GPCR signaling due to the mechanically stressed cytoskeleton. More recent studies utilizing cyclic mechanical stretch on A549 lung adenocarcinoma cell lines revealed that cells activate Wnt/β-catenin and tumor necrosis factor-alpha (TNF-α) inflammation pathways in response to this mechanical stimulus, leading to an increase in cell invasion after stretching (Chen *et al.*, 2023).

Overall, in the primary niche, tumor cells are exposed to a wide variety of mechanical stimuli not found in healthy tissue. Many of these stimuli on their own can irreversibly alter cell behavior, especially in terms of proliferative ability, migratory capacity, traction force development, and directional persistence. This mechanical “boot camp” that pre-metastatic cells are exposed to within the primary tumor niche enables them to more effectively navigate and adapt to even more challenging physical microenvironments encountered in the subsequent migratory niche.

## III. ON THE ROAD: THE PHYSICAL MICROENVIRONMENT OF THE MIGRATORY NICHE

In contrast to the relatively stable microenvironment characterizing the primary tumor site, the physical landscape encountered during the migratory phase presents a distinct array of mechanical challenges. As proliferating cells break free of the primary tumor microenvironment and invade into the surrounding stromal tissue, often led by a single leader cell, they experience a diverse range of physical conditions. To move through these environments, they must undergo confined migration, navigating through highly constricted interstitial spaces and microtracks either inherent to the structure of the interstitial space or created by the secretion of matrix metalloproteinases. Studies utilizing intravital microscopy have quantified the dimensions of these interstitial spaces by directly observing cancer cells migrating through narrow tracks less than 10 *μ*m in diameter and extending over 150 *μ*m in length (Weigelin *et al.*, 2012; Ilina *et al.* 2020). This high degree of confinement can enhance the invasive behavior of cancer cells by triggering the mesenchymal-to-amoeboid transition, reorganizing the cytoskeleton and speeding up cell migration (Tse *et al.*, 2012; Alessandri *et al.*, 2013; Holle *et al.*, 2019; Mosier *et al.*, 2019; and Wang *et al.*, 2019). The nucleus also plays a key role in confined migration, as it must deform greatly during this process. Reducing nuclear stiffness via trichostatin A treatment in MDA-MB-231 cells led to an increase in the invasion depth through dense collagen matrices (Fischer *et al.*, 2020). Furthermore, the nucleus has been shown to act as a “ruler” for detecting spatial confinement, as nuclear deformation past a precise threshold can activate cellular contractility mechanisms (Lomakin *et al.*, 2020). This degree of deformation can be to an extreme degree, to the point that nuclear damage has been correlated with confinement levels (Denais *et al.*, 2016; Irianto *et al.*, 2017). Notably, heightened confinement has also been implicated in fostering resistance to conventional cancer therapeutics, adding an additional layer of complexity to the challenges posed by the physical microenvironment during the migratory phase (Shen *et al.*, 2021).

To support the growth of the proliferating tumor, new vascular networks are often generated via the process of neoangiogenesis. These newly formed vessels are typically leaky, leading to a significant increase in the fluid pressure within the interstitium (Rohde *et al.*, 2000; Stohrer *et al.*, 2000). The magnitude of this pressure increase and the resultant interstitial flow can alter cancer cell migration patterns, allowing them to migrate toward the direction of the flow. This flow often drains into vasculature, providing a potential escape route for cancer cells into circulation (Shieh *et al.*, 2011). Fluid flow and its associated shear force has also been shown to directly detach tumor fragments, allowing them to navigate the interstitial niche more effectively (Tran *et al.*, 2018). When exposed to flow, many cancer cells develop resistance to shear force, enabling their continued proliferation and preparing them for the high shear environment of the vasculature (Yoo *et al.*, 2007). The diverse mechanical cues present within the extracellular matrix of the primary and migratory niches can directly promote proliferation and enhance the invasiveness of cancer cells, which together provide an additive effect that facilitates their progression through the stages of the metastatic cascade. After navigating this environment, the pivotal challenge for these cancer cells lies in their ability to successfully enter and survive the circulatory system, a high shear stress environment in which they can no longer engage an extracellular matrix.

## IV. LETTING GO: THE ADHERENT-TO-SUSPENSION TRANSITION

Metastasizing cancer cells must eventually break into the circulatory system, undergoing transendothelial migration in a process known as intravasation. In circulation, they must navigate through the challenging microenvironment of the bloodstream, which is primarily characterized by high magnitudes of shear forces generated by blood flow and the confining architecture of narrow capillaries. In response to the shear forces within the bloodstream, less adhesive cells within the heterogenous population of cancer cells demonstrate heightened migratory and invasive capabilities (Fuhrmann *et al.*, 2017). Subsequent movement into capillary beds, which presents both shear and confinement to suspended cells, has been found to enhance proliferative capability and resistance to chemotherapy in circulating tumor cells (Jiang *et al.*, 2023).

There are also many factors that contribute to successful intravasation, including other cells in the microenvironment, proteases, signaling molecules, and the environmental conditions of the tumor and the vasculature (Chiang *et al.*, 2016). Once inside the circulatory system, there is no extracellular matrix for them to engage, which can induce anoikis, or anchorage-dependent cell death. Importantly, in order for circulating tumor cells (CTCs) to survive, they must successfully undergo the adherent-to-suspension transition (AST) in order to overcome anchorage dependency and develop a resistance to anoikis.

A recent study sheds light on the dynamic molecular regulation of this transition. Four hematopoietic factors (IKZF1, NFE2, BTG2, and IRF8) whose ectopic expression in adherent cells induces a reprogramming of anchorage dependence were identified (Huh et al., 2023). This reprogramming is achieved through two mechanisms—a YAP/TEAD-dependent mechanism that leads to a loss of cell–matrix interaction and a resistance to anoikis that is achieved via HBA1/2-mediated suppression of lethal reactive oxygen species. Partial induction of AST can also be achieved at low stiffness (10 kPa and 40 kPa) along with YAP-TEAD suppression. Furthermore, these factors were shown to be highly expressed in circulating tumor cells compared to primary tumor cells in breast cancer patients. The reversal of this cell state transition is also important when cells exit circulation, undergo extravasation, and establish colonies in new niches. In this process, cells accumulate lamin A/C in a process mediated by the disruption of the cytoskeleton during the suspension state. This lamin A/C accumulation then promotes cell reattachment and enhanced adhesion (Zhang and Lv, 2017).

The phenomenon of adherent-to-suspension transition is emerging as a novel and exciting facet of cancer research, particularly noteworthy for its pivotal role in the metastatic cascade. Recent studies, exemplified by Huh *et al.* and Zhang *et al.*, have offered valuable insights into the biological regulation of this transition, but many opportunities, especially in the mechanosensitive regulation of this process, remain. These cell state transitions, and the progression from integrin-mediated attachment to long-term survival in suspension and back again, pose interesting and provocative questions on the nature of cytoskeletal-mediated mechanical memory in cancer cells.

## V. A NEW STRONGHOLD: METASTATIC NICHE MECHANICS

The metastatic niche refers to secondary sites where circulating tumor cells colonize and form a new tumor. This process of colonizing the metastatic niche is extremely harsh for the survival of these CTCs as the mechanical microenvironment is already well established. Hence, CTCs have to fit into the metastatic niche by either competing with native cells or by creating their own new niche (Zhuyan *et al.*, 2020). Given that less than 0.01% of circulating tumor cells successfully establish themselves at a new site, the mechanical cues at this location play a crucial role in the selection and survival of these cells (Tremblay *et al.*, 2008).

The “seed and soil hypothesis,” initially formulated by Stephen Paget in 1889, has greatly influenced the field of cancer metastasis and remains relevant to modern ideas of mechanical memory during cancer metastasis (Paget, 1889). This hypothesis emerged as a response to the observation that certain types of cancer exhibit a distinct preference for particular secondary locations within the body. For instance, clinical studies have consistently demonstrated that breast cancer tends to metastasize to the bone, brain, liver, and lung, while prostate cancer frequently displays a preference for the lymph nodes and bone (Edlund *et al.*, 2004; Nguyen *et al.*, 2009).

Although the connectivity of vasculature allows the CTCs to have access to all organs, metastatic sites only develop in select organs. The fundamental premise of the “seed and soil” hypothesis centers on the observation that the microenvironment of the primary and secondary tumor sites is similar. This similarity creates an environment that is both mechanically and biochemically conducive to the survival and growth of metastasizing tumor cells (Er *et al.*, 2018; Piaseczny *et al.*, 2016). When cancer cells encounter a familiar set of mechanical cues in the secondary location, they can more effectively establish themselves without the need to adapt to an entirely novel set of mechanical challenges.

This is supported in various *in vitro* studies that demonstrate how cancer cells exhibit higher rates of proliferation and invasiveness when the mechanical microenvironment at the secondary site closely resembles that of the primary tumor site (Kim *et al.*, 2009). In fact, it is common for cancer cells to recolonize the primary tumor site in a process termed “self-seeding,” often amplifying the aggressiveness of the tumor (Kim *et al.*, 2009). This preference for specific metastatic sites is intricately linked to actomyosin contractility. When the rigidity and mechanical characteristics of the microenvironment closely resemble those of the site of metastasis, cells are optimally primed for navigation and proliferation in the new location (McGrail *et al.*, 2015). These observations form the basis of our current understanding of mechanical memory in the context of cancer metastasis.

## VI. ENGINEERING APPROACHES TO TEST MECHANICAL MEMORY OF CANCER

**FIG. 2.**
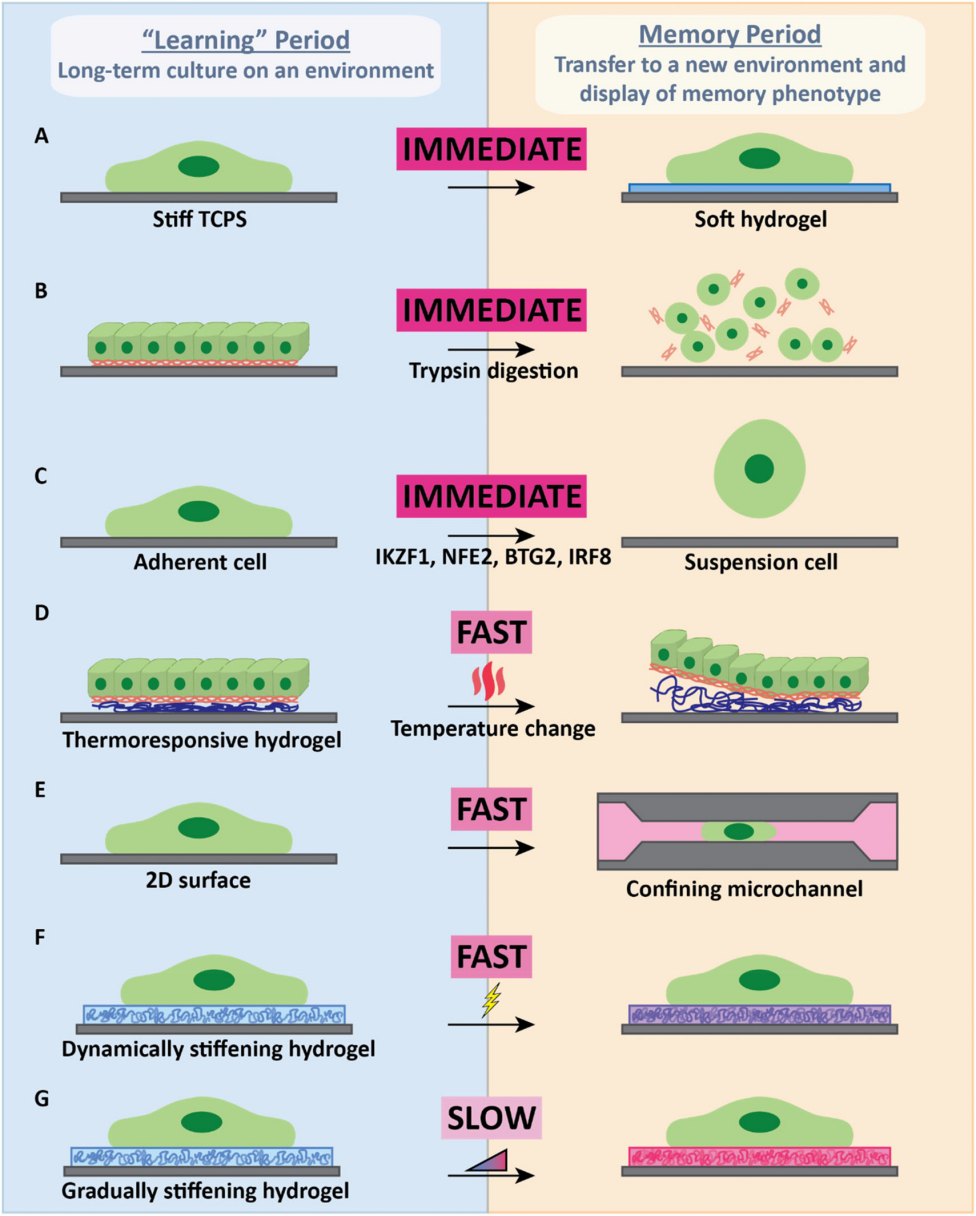
Bioengineering methods used to test mechanical memory. Methods used to test mechanical memory requires a “learning” or dosing period where cells are exposed to the mechanical cues of an environment. Subsequently, the mechanical cues are changed by transferring the cells to a new environment, which can occur at desired time scales. The display of mechanical memory phenotypes is observed in this new environment to determine if memory is permanent or plastic. Mechanical memory as a function of dynamic changes in mechanical cues has been observed in setups such as (a) transfer of stiff tissue culture polystyrene (TCPS)-dosed cells onto soft hydrogels using cell harvesting methods, which can include (b) broad-spectrum enzyme digestion (e.g., trypsin), (c) triggering of AST transition with hematopoietic factors (e.g., IKZF1, NFE2, BTG2, and IRF8), and (d) utilization of thermoresponsive hydrogels that induce detachment in response to a rapid temperature change. Other setups used to study mechanical memory-based behavioral changes on a slower timescale include (e) the use of cell migration to bring cells from one microenvironment into another (typically from 2D to 3D), (f) hydrogels capable of dynamic stiffening in response to a rapid stimulus (i.e., hours to stiffen), and (g) hydrogels that stiffen gradually due to cross-linking dynamics (i.e., days or weeks to stiffen).

The metastatic journey is marked by a plethora of microenvironmental mechanical stimuli, fueling diverse opinions on which cue or combination of cues constitutes the key features of mechanical memory crucial for conferring a selective advantage to cancer cell metastasis. In order to systematically test these cues, a wide variety of engineering approaches have been employed to better understand the role of mechanical memory during cancer metastasis (Fig. 2).

Hydrogels, with their easily tunable mechanical properties, have been used widely to explore stiffness as a mechanical memory cue, allowing researchers to expose cells to a spectrum of stiffness ranges that closely mimic physiological values. These engineered environments can also be modified with different compositions of ECM proteins that can enhance or block stiffness-induced proliferation (Sawicki *et al.*, 2019). Serial passaging of breast cancer cells on soft polyacrylamide substrates revealed that cancer cells adapt to compliant surfaces by improving their attachment and increasing their cell area (Syed *et al.*, 2017). While this proves that prolonged mechanical exposure to a compliant ECM impacts cancer cell phenotype, whether this response is genetic, epigenetic, or phenotypic remains an open question. The use of different stiffnesses in time series has also been employed to test mechanical memory-based cellular responses [Fig. 2(a)]. Plating breast epithelial MCF10A cells on soft or stiff polyacrylamide hydrogels for a specific duration, followed by replating on the opposite stiffness, demonstrated that stiff dosing led to cells retaining nuclear YAP and migrating at a higher speed. Additionally, when these stiff-dosed cells were allowed to invade into three-dimensional collagen matrices, they transferred their mechanical memory to the matrix by remodeling and aligning the collagen fibers to reduce tension and promote further cell invasion (Almeida *et al.*, 2023; Nasrollahi *et al.*, 2017). This force modulation has also been observed in the context of secondary niche preference, as stiff dosing was shown to increase cellular traction force and confer a selective preference for bone metastasis *in vivo*, a process mediated by prolonged expression of the osteogenic transcriptional factor RUNX2 (Watson *et al.*, 2021). Likewise, using 3D printed breast-to-bone *in vitro* models that mimic the composition and stiffness of cancer tissue and the metastatic niche, dosing on a higher hydrogel stiffness results in increased migration and invasion of MDA-MB-231 cells following an increased expression of osteolytic factors PTHrP and IL-6 (Shah *et al.*, 2023). In three-dimensional systems, stiff mechanical environments cause cells to display a more wrinkled nuclear envelope, accompanied by increased lamina-associated chromatin. Consequently, this leads to more accessible chromatin sites, facilitating the expression of genes associated with tumorigenicity (Stowers *et al.*, 2019). Mechanical memory is not just responsible for triggering invasive cells to be more invasive; it has also been shown to push noninvasive cells to an invasive state. Typically, noninvasive oral squamous cell carcinoma cells that were dosed on stiff niches for prolonged periods of time were shown to acquire an invasive phenotype that can persist even after the niche is changed. This stiffness-dependent contractility was mediated by AKT signaling, and the persistence of the phenotype was controlled by focal adhesion kinase (FAK) activity (Matte *et al.*, 2019; Moon *et al.*, 2023). However, not every mechanosensitive cell is capable of exhibiting hallmarks of mechanical memory, as was found with mammary epithelial cells on gradually stiffening hydrogels (Ondeck *et al.*, 2019). It is also worth noting that transformed cells often exhibit reduced sensitivity to substrate stiffness cues due to dysregulated mechanosensitive signaling pathways (Kostic et al., 2009, Yang *et al.*, 2020), which likely also blunts their ability to integrate mechanical signals into their mechanical memory.

Given the complexity of the metastatic microenvironment, some have started to explore dosing with alternative mechanical cues beyond substrate stiffness. Dosing breast cancer cells with elevated levels of extracellular fluid viscosity was found to increase confined migration *in vitro*. This behavior was recapitulated *in vivo*, with enhanced migration and colonization of viscosity-dosed cells observed in zebrafish, chick embryo, and mice, in a process mediated by Hippo pathway-dependent TRPV4 expression (Bera *et al.*, 2022). Mechanical cue like the increase in fluid pressure in the tumor environment is also a feature of disease progression. Many engineering methods have already been developed to look into cues such as hydrostatic pressure and its effect on cancer cells, and these methods have been nicely reviewed by Purkayastha *et al.* (2021). These engineered systems can be used to determine if pressure cues affect mechanical memory and pushes the field toward less explored mechanical memory cues that do not rely on cell–ECM adhesions.

While the presentation of matrix-associated cues, especially in three dimensions, is an opportunity for bioengineered systems, cancer metastasis is inherently a process that occurs in four dimensions. To investigate the temporal dimension of mechanical memory, models have been developed aimed at capturing the dynamic alterations in memory retention corresponding to varying dosing durations (Price *et al.*, 2021). Experiments probing mechanical memory typically look for rapid cellular responses to mechanical signals, often occurring within minutes to hours. This rapid response aligns with mechanosignaling processes and is reinforced through transcription and translation mechanisms. In contrast, the timescale associated with the development and dissipation of mechanical memory is much longer, extending from days to weeks depending on the microenvironmental history. These prolonged timelines are attributed to shifts in stable chromatin states prompted by nuclear tension, epigenetic modifications, and alterations in post-transcriptional regulation. The heightened levels of transcriptomic, epigenetic, and proteomic reinforcement collectively contribute to the gradual stabilization of cellular phenotypic features and the slow dissipation of mechanical memory. Utilizing tools that are capable of dynamically changing substrate stiffness on a short-term [Fig. 2(g)] [e.g., magnetic hydrogels (Chen *et al.*, 2021; Yan *et al.*, 2022), chemically induced stiffening (Li *et al.*, 2023), and other stimuli-responsive hydrogels (Abdollahiyan *et al.*, 2020)] and long-term [Fig. 2(f)] [e.g., matrix metalloproteinase-degradable hydrogels (Yu *et al.*, 2016) and time-dependent stiffening hydrogels (Guvendiren and Burdick, 2012; Young and Engler, 2011)] will emerge as robust engineering strategies for comprehending the intricacies of mechanical memory dynamics.

With each passing year, a growing number of new and diverse engineering approaches are developed with the aim of deciphering mechanical memory. As the field continues to innovate, it will be important to keep the physiological conditions in sight, especially with respect to timescale. While dynamically stiffening systems capable of rapid, seconds-scale changes in substrate stiffness may provide fundamental insights in how cells respond to their environment, they may not be the best tool to study the months-long process of primary niche stiffening. Long-term stiffening systems allowing for much longer and gradual changes in stiffness can be developed to model this response, but they would not be useful in understanding metastasis to a secondary niche, which requires an adhesion-to-suspension transition. To fully understand this process, open questions remain about the degree to which mechanical memory is altered by a standard treatment with broad-spectrum enzymes like trypsin for passaging and whether alternative cell detachment methods can alter mechanical memory retention. It is noted, however, that trypsin digestion does not completely eliminate mechanical memory and has been utilized on multiple occasions in mechanical memory experiments where cells are replated from stiff to soft substrates (Balestrini *et al.*, 2012; Yang *et al.*, 2014) [Fig. 2(b)]. This provides an opportunity for understanding the implications of cell-driven detachment on maintenance of memory and the emerging mechanosensitive pathways underlying the adhesion-to-suspension transition [Fig. 2(c)]. As mechanical memory is inherently influenced by the duration of exposure to the mechanical cue, whether the duration spent while cells are in suspension erases the mechanical memory of their stiffness priming and elicits new memory due to the shear forces remains to be explored. Ultimately, bioengineers exploring this space should continue to draw inspiration from the physiology as they develop new *in vitro* tools, especially with respect to the dynamic nature and diverse timescales of the metastatic cascade *in vivo*. As they do so, the opportunities for leveraging advances in our understanding of mechanical memory to translational clinical strategies will become more numerous and impactful.

## VII. TRANSLATIONAL APPROACHES TO UTILIZING MECHANICAL MEMORY

In the pursuit of advancing cancer therapeutics, the translation of fundamental scientific insights into tangible clinical applications is paramount. Understanding how to harness advances in our understanding of mechanical memory into practical benefits is a critical endeavor, particularly with respect to the development of bioengineered systems that integrate the principles of mechanical memory into their design.

Many diverse biomaterials have emerged through translational research on mechanical memory, showcasing their potential impact on diverse cellular behaviors. Notably, investigations using dynamically softening hydrogels have also revealed intriguing interdisciplinary findings. Culture on substrates that are dynamically softened by matrix metalloproteinases not only facilitates the storage of mechanical memory, but also results in a weaker malignant potential when cells are transferred to stiffer surfaces (Hu *et al.*, 2021). This study implies that by directly targeting stiffness at the primary tumor, we can leverage the storage of mechanical memory by cancer cells even when they later experience a stiffer mechanical environment. Beyond cancer cells, thermoresponsive hydrogels with tunable stiffness have been shown to be beneficial for fibroblasts, allowing the harvesting of mechanically dosed cells with phenotypes conducive to wound healing and skin recovery *in vivo* (Fan *et al.*, 2017) [Fig. 2(d)]. The utilization of an alginate-based hydrogel capable of dynamically switching its mechanical properties was used to identify microRNA miR-21 as a sensor for long-term mechanical memory in human mesenchymal stem cells (Wei *et al.*, 2020). By modulating miR-21 levels in stiff-primed mesenchymal stem cells, mechanical memory phenotypes can be effectively erased, resensitizing cells to new substrate mechanics and priming their differentiation toward desired lineages (Li *et al.*, 2017). The effective rewiring of mechanical memory by modulating the miR-21 levels in CTCs has potential to be utilized in future cancer therapy.

Alterations to the extracellular environment not only influence fast cellular responses but also contribute to the establishment of memory-biased cues for newly seeded cells. For example, ECM remodeling facilitated by cancer-associated fibroblasts can induce changes in the pro-angiogenic signaling mechanisms of newly seeded cells, showing that remodeling can impart long-term changes in cells (Wang *et al.*, 2017). Furthermore, recent work has shown that highly mineralized bone ECM derived from mice can impact the growth and behavior of newly seeded human breast cancer cells, namely, reduced tumor growth and an upregulation of anti-metastatic genes (Choi *et al.*, 2023). This implies that the memory of modified ECM persists and continues to influence cellular responses, hinting at the potential use for therapeutic purpose in inducing mechanical priming in cancer cells via the introduction of ECM-modifying drugs that impart memory-based long-term cues to prevent metastasis of cancer cells.

These studies not only shed light on the potential applications of mechanical memory as a parameter for bioengineered *in vitro* cell culture systems, but also emphasize its role as a crucial factor in manipulating cell–ECM interactions. While translational research in the context of cancer metastasis is still in its early stages, the implications of mechanical memory offer promising avenues for leveraging these interactions to enhance human health. As this field continues to evolve, the integration of mechanical memory into biomaterial design stands poised to impact a wide variety of therapeutic strategies by exploiting the dynamic interplay between cells and their microenvironment.

## VIII. FUTURE OPPORTUNITIES IN LEVERAGING MECHANICAL MEMORY IN A BIOENGINEERING CONTEXT

While not an entirely new concept, the appreciation of mechanical memory has surged in recent years, gaining recognition as a critical factor in the understanding of cancer biology and treatment. However, this acknowledgment remains a relatively recent development, and many studies still tend to overlook the four-dimensional influence of mechanical memory on cancer cell behavior. Remarkably, there has been a notable shift in the collective awareness of researchers toward the importance of incorporating mechanical memory considerations into their functional assays and biological inquiries. Increasingly, scientists are recognizing the need to go beyond conventional mechanosensing experiments, which often neglect the lasting effects of prior mechanical activation caused by prolonged cell culture on stiff surfaces. The growing realization that prolonged exposure to rigid culture surfaces profoundly impacts cancer cell behavior is driving a paradigm shift in the experimental design. Future functional studies are poised to adopt innovative approaches that involve pretreating cancer cells on compliant surfaces, erasing the persistent memory of glass or tissue culture plastic and mirroring the stiffness range of physiological tumor microenvironments more accurately. This shift promises to elevate the fidelity of diagnosis and drug testing assays that rely on *in vitro* cells resembling their *in vivo* counterparts. The field is also becoming more attuned to the nuanced dynamics of cancer cells navigating through the interstitial space—a key mechanical memory cue often overlooked in previous studies. This confined migration, occurring upstream of many other processes in the metastatic journey including the adherent-to-suspension transition and the colonization of the secondary site, deserves consideration as a critical, if not primary, factor influencing mechanical memory [Fig. 2(e)]. Pre-dosing cells to a particular environment prior to confinement can allow us to determine how cells at the primary niche gain mechanical memory and how this mechanical memory impacts their ability to undergo confined migration. Alternatively, confined migration itself can also be a mechanical memory cue that can result in difference in a cell's metastatic potential. Early findings into this idea showed that MDA-MB-231 single cells exiting short 10 *μ*m-length confining microchannels tended to form distinct two protrusions at the leading edge of the cell and the memory of this protrusion formation can persist up to 60 min of migration (Zhang *et al.*, 2021). Memory of highly confined regions has also been shown to influence cell migration speed as they exit to regions of less confinement, resulting in faster migration of cells fueled by mitochondria activity at the cell front (Mosier *et al.*, 2023). This further emphasizes that mechanical memory in the context of confined migration is an exciting and relevant field that is worthy of more in-depth exploration.

## Data Availability

Data sharing is not applicable to this article as no new data were created or analyzed in this study.
